# Valproic acid autoinduction: a case-based review

**DOI:** 10.1186/s40345-021-00232-6

**Published:** 2021-09-01

**Authors:** Sean Bennett, Mujeeb U. Shad

**Affiliations:** 1 Samaritan Health Services, Corvallis, OR USA; 2 University of Nevada Las Vegas, Las Vegas, NV USA; 3 Touro University Nevada, Las Vegas, NV USA; 4 Valley Health System, Las Vegas, NV USA

**Keywords:** Valproic, Acid, Autoinduction, Case-based, Review

## Abstract

Although valproic acid (VPA) induces the metabolism of multiple other drugs, the clinical reports of VPA autoinduction are rare. A comprehensive literature search yielded only one published case series, which provided the rationale to conduct a review of the published cases along with a new case of VPA autoinduction. Although there may be myriad of reasons for lack of published cases of VPA autoinduction, potential underreporting may be one of the core reasons. Lack of understanding into the highly complex metabolism of VPA may also make it difficult to recognize and report VPA autoinduction. However, it is important to mention that in addition to autoinduction increased elimination of VPA may be mediated by several pharmacokinetic (PK) factors, such as drug interactions, genetic polymorphisms of metabolic enzymes, and protein displacement reactions. As VPA is metabolized by multiple metabolic pathways, the risk for drug interactions is relatively high. There is also a growing evidence for high genetic inducibility of some enzymes involved in VPA metabolism. Protein displacement reactions with VPA increase the biologically active and readily metabolizable free fraction and pose a diagnostic challenge as they are usually not requested by most clinicians. Thus, monitoring of free fraction with total VPA levels may prevent clinically serious outcomes and optimize VPA treatment in clinically challenging patients. This case-based review compares the clinical data from three published cases and a new case of VPA autoinduction to enhance clinicians' awareness of this relatively rare but clinically relevant phenomenon along with a discussion of potential underlying mechanisms.

## Introduction

Valproic acid (VPA) is one of the oldest and most frequently prescribed drugs for epilepsy, bipolar disorder, and migraine prophylaxis (Peterson and Naunton 2005). This is one of the few psychotropic drugs that have fairly well-defined therapeutic levels that range from 50 to 100 µg/mL for epilepsy and 80 to 125 µg/mL for bipolar disorder (Patsalos et al. 2008). These therapeutic levels are usually achieved at VPA doses well under 4000 mg/day. However, VPA doses required to achieve recommended therapeutic levels vary significantly across patients, which could be due to its highly complex metabolism. Valproic acid is metabolized by both phase-I and phase-II enzyme systems as well as fatty acid *β*-oxidation. Although rarely reported, VPA autoinduction is a clinically-significant phenomenon in a high-risk patient population with bipolar disorder and epilepsy as sub-therapeutic VPA levels may result in clinically serious outcomes, such as suicide and seizures, respectively. A comprehensive literature search yielded only one published case series reporting three cases of VPA autoinduction (Jackson et al. 2015), which could be due to potential under-reporting, lack of understanding into the complex nature of VPA metabolism as well as lesser recognition of drugs as inducers than inhibitors (de Leon 2015a, b). Finding the current case of VPA autoinduction provided us the rationale to conduct a review of the published cases along with the new case in order to analyze and compare clinical data and increase clinicians’ awareness into VPA autoinduction. This case-based review also provided an opportunity to discuss various pharmacokinetic mechanisms underlying VPA autoinduction to minimize seriously adverse outcomes and optimize clinical outcomes in challenging patients.

## Methods

The current case of the bipolar patient hospitalized for inpatient treatment was identified after he required unusually high dose of VPA to achieve therapeutic levels. A comprehensive literature search yielded only one case series comprised of three patients who required unusually high VA doses to be effective. The current case was added to the three cases of VPA autoinduction in the published case series to conduct a case-based review. The literature search was conducted in MEDLINE/PubMed, EMBASE, CINAHL, PsychINFO without any restrictions. The following search terms were employed for this search: ((‘valproic acid’ OR ‘valproate’ OR ‘VPA’) AND (‘autoinduction’ OR ‘auto-induction’ OR ‘induction’ OR ‘inducibility’)). A total of 13 records were found of which four records included both search terms and one record documenting VPA autoinduction in three patients in a case series. All VPA levels were trough levels obtained at the steady state, including the current case. The concentration/dose (C/D) ratio for the current case was calculated using the same methods and rationale as described in the published case series (Jackson et al. 2015). Briefly, the VPA level was divided by the VPA dose and multiplied by 1000 to make the ratio numerically clear.

## Results

As can be seen from the Table 1, all reviewed cases were Caucasian males, with a mean age (SD) of 55.25 ± 14.17, receiving a formulation of VPA for various diagnoses, including bipolar disorder, schizophrenia, tuberous sclerosis, and traumatic brain injury. However, the clinical data for three published cases were collected over several years of inpatient treatment as compared to clinical data for the current subject over a much shorter hospital stay of 22 days. The current patient was admitted for severe traumatic brain injury, methamphetamine use, and bipolar symptoms, such as racing thoughts, lack of sleep, increased energy, and pressured speech, but without any other psychiatric or neurological disorders. VPA levels for all cases were trough levels obtained at a steady state using the same clinical laboratory. However, in contrast to several years of data collection for the three published cases, the VPA levels in the current case were obtained only over 22 days. The normal therapeutic ratio of VPA C/D ratio × 1000 ranged between 112 and 132 µg/mL.

**Table 1:** Relationship between age, weight, valproic acid (VPA) formulation, daily VPA dose, average VPA levels, and concentration/dose (C/D) ratio × 1000 mean ± SD and range across cases

Cases	Age	Wt. (kg)	Diagnosis	Hospital Duration	Formulation	VPA dose (mg/day)	VPA level (μg/mL)	C/D ratio × 1000^a^	No. of VPA Levels	Mean C/D ratio × 1000: mean ± SD, range^#^	Comments
Case 1^*^	34	85	Schizophrenia Seizure disorder	> 3 years	VPA	5250	87	17	44	15 ± 2.6, range = 10–21	Significantly lower C/D ratio than the normal range between 112 and 132 µg/mL, suggesting faster VPA metabolism
				< 1 year	DVPS	2000	78	39	7	39 ± 6.3, range = 28–48	Higher C/D ratio with DVPS than VPA in case 1, suggesting significantly slower DVPS metabolism than VPA
Case 2^*^	66	90	Bipolar Disorder Substance Use disorder	8 weeks	DVPS	4000	67	17	10	25 ± 5.6, range = 17–33	Significantly lower C/D ratio than the normal range between 112 and 132 µg/mL, suggesting faster VPA metabolism
Case 3^*^	70	71	Tuberous Sclerosis	> 4 years	DVPS	10,500	10	12	137	8 ± 3.5, range = 3–20	Lowest C/D ratio of all cases, suggesting robust autoinduction with DVPS
New Case	51	71	Bipolar disorder Methamphetamine use disorder Recent Traumatic Brain Injury	22 days	VPA	5000	98.8	19.8	7	18.6 ± 2.9, range = 13–20	Similar C/D ratio to case 1 & 2, suggesting similar rate of autoinduction with VPA

*Reference = Jackson et al. (2015) VPA: valproic acid; DVPS: divalproex sodium; C/D = Mean concentration/dose ratio (therapeutic range between 112 to 132 µg/mL); Number of VPA levels drawn over total hospitalization

^#^Mean C/D ratio × 1000: mean ± SD, range; Calculated from the total number of VPA levels during hospital duration

^a^C/D ratio × 1000; Calculated from the last final VPA dose and the level at that dose

## Brief case histories of previously published cases

### Case 1

A 34-year old Caucasian male with treatment-refractory schizophrenia developed a seizure disorder before he was hospitalized with four antiepileptic medications: carbamazepine, phenytoin, diazepam, and VPA, but all with subtherapeutic levels. It took several months to titrate off these medications, except VPA. The seizures were not adequately controlled until the VPA dose was increased to 5250 mg/day to achieve therapeutic concentrations. However, VPA had to be switched to divalproex sodium (DVPS) after the patient began to complain about the bad taste of the VPA concentrate. This switch led to an unexpected valproate intoxication despite the absence of any other medication changes.

### Case 2

This patient was a 66-year-old Caucasian male with a long history of bipolar disorder and substance use disorder. He was admitted to a psychiatric hospital for bipolar symptoms and psychosis, which was managed by starting 1000 mg/day DVPS. However, he continued to display rapid, pressured speech and flight of ideas, in addition to increased levels of energy, which required a gradual increase in DVPS dose to 4000 mg/day to have an antimanic response with a therapeutic level of around 70 μg/mL.

### Case 3

This patient was a middle-aged Caucasian male with tuberous sclerosis manifested as a seizure disorder and mental retardation. He had been in inpatient treatment at a state psychiatric hospital for about 22 years before he started to have complications from tuberous sclerosis, including a right renal tumor, worsening episodes of ataxia and confusion, and a giant cell astrocytoma. Thus, the patient required a progressive increase in the DVPS dose from a baseline dose of 3375 mg/day to a therapeutic dose of 10,500 mg/day. This dose was maintained until it was found that the patient's physical deterioration was due to astrocytoma, resulting in discontinuation of all medications except phenytoin and a reduction in DVPS from a dose of 10,500 to 7000 mg/day.

### Current case

A 51-year old Caucasian male with past psychiatric history of bipolar disorder and methamphetamine use disorder, who was consulted by Consult-Liaison (C-L) psychiatry following a three-week inpatient surgical hospitalization after the wreckage of his recreational vehicle while under the influence of methamphetamine. Injuries sustained resulted in a skull fracture and intracranial hemorrhage, requiring neurosurgery. Recovery was then complicated by ongoing aggression and agitated delirium, for which CL psychiatry was consulted. The initial efforts were to reduce the use of as-needed medications, discontinue deliriogenic medications, and transition the patient to high dose IV haloperidol to manage aggression. A dose of 156 mg IV haloperidol over 24 h was administered but had to be discontinued due to prolonged QTc interval, with the highest QTc value reaching 560 ms and a return to 420 ms following haloperidol's discontinuation. Three different second-generation antipsychotic medications, benzodiazepines, and opiate pain medications failed to adequately manage patient’s aggression, At this time, a collaborative, multi-team decision was made to intubate the patient and perform a medication washout due to refractory, severe, and persistent agitated delirium. Eventually, valproic acid (Depakene®), quetiapine and risperidone were introduced to manage aggressive and impulsive behavior in a setting of traumatic brain injury (TBI) with a history of possible underlying bipolar mania. Following extubation and washout, the delirium resolved. The patient was alert and able to fully orient and converse; however, he remained agitated and aggressive with staff, demanding to leave against medical advice (AMA). In the meanwhile, repeated VPA dose titrations were required to achieve therapeutic trough levels of 98.8 µg/mL at the steady state at an unusually high total dose of 5000 mg/day. Eventually, the patient stabilized and was discharged on day 22 of hospitalization. Of note, risperidone was discontinued after the 18th day of VPA initiation to reduce polypharmacy.

## Discussion

As can be seen from the Table 1, similar to the prior cases, the new case (case 4) required aggressive titration of VPA dose to achieve therapeutic VPA levels required for bipolar disorder (80–125 µg/mL) (Peterson and Naunton 2005). Although it took several years for the published cases to attribute the need for unusually high VPA doses to autoinduction (Jackson et al. 2015), it took only a few days to do so for the current case due to the valuable clinical information provided by the published case series. Since VPA has a highly complex metabolism, an initial discussion of different PK factors involved in VPA autoinduction may be relevant here. About 40% of VPA is metabolized by multiple isoforms of a phase-II enzyme, uridine diphosphate glucuronyl transferase (UGT1A6, UGT1A9, and UGT2B7), (Li et al. 2004; Patsalos and Perucca 2003); while 30% is metabolized via *β*-oxidation, and the rest by multiple phase-I cytochrome P450 (CYP) enzymes (i.e., CYP2A6, CYP2C9, and CYP2C19) (Ghodke-Puranik et al. 2013; Perucca et al. 2006). It is interesting to note that two of the three UGT isoforms (i.e., UGT2B7, UGT1A9) and one of the CYP enzymes (CYP2C9) that metabolize VPA are actually inhibited by VPA (Morris et al. 2000). In this context, inhibition of UGT2B7 is well-documented to elevate lamotrigine levels when co-administered with VPA (Wang et al. 2016) by 2–3 folds (Morris et al. 2000) increasing the risk for dose-dependent life threatening skin rashes associated with lamotrigine. This is despite some animal studies reporting increased VPA glucuronidation (Fisher et al. 1991) and *β*-oxidation (Fisher et al. 1991; McLaughlin et al. 2000) but with long-term use of VPA. Another study (McLaughlin et al. 2000) found at least 3 weeks delay in mild VPA autoinduction with a low-dose VPA mediated by the *β*-oxidation but not UGT. The delayed enzyme induction probably does not explain the relatively fast autoinduction observed in reviewed cases. However, there are many other PK factors that can also alter VPA plasma levels, including drug interactions, protein displacement reactions, genetic polymorphisms, and activation of nuclear receptors.

In terms of drug interactions, VPA is known to elevate plasma levels of only a few drugs, most notably lamotrigine (Wang et al. 2016; Morris et al. 2000). However, VPA is more frequently reported to increase the metabolism of multiple co-administered drugs, such as aripiprazole (Citrome et al. 2005), clozapine (Cerveny et al. 2007; Finley and Warner 1994; Longo and Salzman 1995; Diaz et al. 2014), norclozapine, felbamate (Hooper et al. 1996), irinotecan (Jong et al. 2007), and olanzapine (Bergemann et al. 2006; Haslemo et al. 2012). The relatively rare reports of VPA autoinduction suggest that the VPA-induced induced enzymes may not be relevant in VPA metabolism. Regardless, the high level of long-term polypharmacy in the published cases was not observed in the current subject, who was only treated with two concomitantly administered medications (i.e., 4 mg/day of risperidone & 950 mg/day of quetiapine). Both these medications were co-administered at the same dose for 18 out of 22 days of hospitalization, and none has been reported to significantly interact with VPA. Most of the other concomitant drugs in the published cases are not known to significantly alter VPA metabolism with the only exception of phenytoin in case 3. Phenytoin is a robust inducer for enzymes, including UGT and may have contributed to the VPA autoinduction (Zaccara et al. 2014).

The protein displacement reactions may also enhance VPA metabolism, which may sometimes be confused with autoinduction. Valproic acid is highly protein-bound drug, which can saturate protein binding sites at levels ≥ 50 µg/mL (Jackson et al. 2015), thereby increasing the biologically active and readily metabolizable free fraction (Dutta et al. 2007; Kodama et al. 2001). A similar increase in free fraction may occur if VPA is displaced by more potent protein-bound drugs, such as ibuprofen (Lana et al. 2016a, 2016b). Thus, dose-dependent protein saturation is one of the many non-inducing factors that can magnify VPA autoinduction. Valproic acid, in turn, may displace phenytoin, which is a lesser protein bound drug (Lai and Huang 1993; Perucca et al. 1980), thereby decreasing its plasma levels (Wen et al. 2001). This decrease is often counterbalanced by an increase in phenytoin levels via VPA-induced inhibition of CYP2C9 that metabolizes phenytoin (Franco and Perucca 2015). This observation is consistent with unchanged phenytoin levels in case 3. Since albumin is the plasma protein that binds VPA (Hermida and Tutor 2005; VandenBerg et al. 2017), any change in albumin levels may also alter VPA levels. Thus, an increase in VPA free fraction has been reported in patients with hypoalbuminemia due to acute medical stress and renal or liver dysfunction (Hermida and Tutor 2005; VandenBerg et al. 2017). However, increased free fraction from protein saturation is clinically different from that reported with hypoalbuminemia. Unlike protein saturation, any increase in VPA dose in patients with hypoalbuminemia may overwhelm the metabolic pathways to effectively eliminate significantly elevated levels of biologically active free fraction, thus increasing the risk for VPA toxicity in the absence of a notable increase in total VPA levels (Hermida and Tutor 2005). This underscores the need to obtain free fraction levels in addition to the total VPA levels to guide critical VPA dose adjustments in clinically challenging cases (Maat et al. 2011). A simple subtraction of free fraction from the total VPA levels may help determine protein-bound VPA levels to distinguish between protein saturation and hypoalbuminemia.

Genetic polymorphisms in enzymes that metabolize VPA may explain the high inter-individual variability in VPA levels (Methaneethorn 2018; Ding et al. 2015). The most significant inducible variants for UGT1A6 are 541A > G and 552A > C), which have been associated with increased metabolism of VPA in children (Guo et al. 2012; Hung et al. 2011; Wang and Zhou 2016). A relatively recent metanalysis supported the inducibility of the two UGT1A6 variants in other age groups as well (Kim and Kim 2019). Another study reported a third inducible UGT1A6 variant (i.e., 19T > G) as well as a CYP2C9 variant in VPA-treated patients with severe traumatic brain injury (Sun et al. 2017). Tanner and Tyndale (2017) found a second inducible CYP enzyme, 2A6 to further contribute to the significant inter-individual variation in VPA levels (Tanner and Tyndale 2017). Inducible variants of UGT2B7 (i.e., G211T and C161T) were also reported to require VPA dose adjustments for therapeutic effects in a metanalysis (Wang et al. 2018). Since UGT variance was not assessed in the reviewed cases, the effects of UGT polymorphism(s) on VPA autoinduction cannot be assessed. Nevertheless, genetic testing for CYP2C9 and CYP2C19 that metabolize VPA (Jiang et al. 2009) ruled out any dysfunctional alleles for these enzymes in the published case series (Jackson et al. 2015).

Another potential mechanism for VPA autoinduction could be the activation of a nuclear receptor, peroxisome proliferator-activated (Kallen 2004; Lampen et al. 1999; Werling et al. 2001), which has been associated with induction of UGT enzymes (Fisher et al. 1991; Luci et al. 2006). However, it is unlikely that activation of other nuclear receptors associated with increased expression of CYP3A4 and P-glycoprotein (P-gp) (Cerveny et al. 2007) could have contributed to the VPA autoinduction as VPA is not a substrate for either of the two. This is in contrast to another antiepileptic drug, carbamazepine, which is associated with robust autoinduction as it increases the expression of CYP3A4 and P-gp, for which it is a substrate (Lutz et al. 2018).

This review has several limitations including the retrospective nature of the clinical data, the lack of VPA free fraction level monitoring, the significant difference in hospital duration between the current and published cases, the lack of post-discharge follow-up and VPA level monitoring for the current case. Nevertheless, this review underscores the clinical significance of not only monitoring total VPA levels, but also the free fraction, to facilitate diagnosis and management of VPA autoinduction.

## Conclusion

The findings from this review are based on a retrospective analysis of only four cases and should be interpreted with caution. However, the clinicians should consider VPA autoinduction in patients with unusually low plasma levels despite conventional VPA doses. It appears that VPA autoinduction is a relatively rare phenomenon probably because of multiple PK factors that may result in or contribute to VPA autoinduction, most notably drug interactions, protein displacement reactions, and genetic polymorphisms in enzymes metabolizing VPA. Until future research addresses the complexities of VPA metabolism, the most logical way to deal with clinically challenging cases is to obtain both total and free fraction VPA levels for safe and effective VPA dose adjustments.

## Data Availability

N/A.
